# Packet-level and IEEE 802.11 MAC frame-level network traffic traces data of the D-Link IoT devices

**DOI:** 10.1016/j.dib.2021.107208

**Published:** 2021-06-11

**Authors:** Rajarshi Roy Chowdhury, Sandhya Aneja, Nagender Aneja, Pg Emeroylariffion Abas

**Affiliations:** a Faculty of Integrated Technologies, Universiti Brunei Darussalam, Jalan Tungku Link, Gadong, Brunei Darussalam; b Faculty of Science, Universiti Brunei Darussalam, Jalan Tungku Link, Gadong, Brunei Darussalam

**Keywords:** Internet of Things: Network traffic, Protocol packet, IEEE 802.11 MAC frame, Probe request frame

## Abstract

With the growth of wireless network technology-based devices, identifying the communication behaviour of wireless connectivity enabled devices, e.g. Internet of Things (IoT) devices, is one of the vital aspects, in managing and securing IoT networks. Initially, devices use frames to connect to the access point on the local area network and then, use packets of typical communication protocols through the access point to communicate over the Internet. Toward this goal, network packet and IEEE 802.11 media access control (MAC) frame analysis may assist in managing IoT networks efficiently, and allow investigation of inclusive behaviour of IoT devices. This paper presents network traffic traces data of D-Link IoT devices from packet and frame levels. Data collection experiment has been conducted in the Network Systems and Signal Processing (NSSP) laboratory at Universiti Brunei Darussalam (UBD). All the required devices, such as IoT devices, workstation, smartphone, laptop, USB Ethernet adapter, and USB WiFi adapter, have been configured accordingly, to capture and store network traffic traces of the 14 IoT devices in the laboratory. These IoT devices were from the same manufacture (D-Link) with different types, such as camera, home-hub, door-window sensor, and smart-plug.

## Specifications Table

**Table:** 

Subject	Computer Networks and Communications
Specific subject area	Network traffic traces (packet-level and frame-level) of the specific purpose D-Link IoT devices
Type of data How data were acquired	Network Traffic Traces Data derived from the experimental testbed of 14 D-Link IoT devices in the Network Systems and Signal Processing (NSSP) laboratory, Universiti Brunei Darussalam (UBD). **Hardware:** Workstation, Laptop, Smartphone, D-Link IoT devices, USB Ethernet adapter, USB WiFi adapter. **Software:** Kali Linux, Ubuntu, Vmware Player, Wireshark, Shell Scripts, pcapfix, etc.
Data format	Raw (pcap files)
Parameters for data collection	IoT devices network traffic traces were collected in two circumstances: autonomous traffic (traffic traces generated automatically without any user interaction with the devices, e.g. network time protocol (NTP) for time synchronization) and activity traffic (traffic traces generated according to users activities, e.g. viewing camera locally or remotely, movement in the range of motion sensor) due to the surrounding environment. All these traffic traces were captured according to the IoT device MAC and access point MAC addresses. Network traffic traces were collected for about five months, from 9^th^ September 2020 to 10^th^ January 2021.
Description of data Collection	Network traffic traces were collected passively through the observation of IoT devices communication traffic, with the IoT devices configured to connect to specific access points. Packet traces were captured on the guest (Kali Linux) operating system (OS) running an access point, while IEEE 802.11 MAC frame data were recorded on the host (Ubuntu) OS monitoring a specific access point (AP). Additionally, network packet traces were captured on the host OS, observing the connected external Ethernet interface. The D-LinkHomeHub device was connected to the host OS through an external Ethernet interface, with some devices, e.g. door-window sensor, camera, utilizing the D-LinkHomeHub as an AP to get Internet services. On both access points, tcpdump was used for data collection.
Data source location	Institution: Universiti Brunei Darussalam Region: Brunei-Muara Country: Brunei Darussalam Latitude and Longitude: 4°58′41.7″N 114°53′50.5″E
Data accessibility	The recorded pcap raw files are available on the Mendeley Data repository, which are publicly available on https://data.mendeley.com/datasets/84cc8grtkt/1

## Value of the Data

• The provided IoT datasets facilitate researchers to evaluate network traffic traces in two levels: the network packets of protocols used by the IoT devices to communicate over the Internet, and the IEEE 802.11 MAC frames used by the IoT devices to connect to an access point in a local area network.
• The datasets will assist researchers from academia and industries to analyze communication patterns of the 14 IoT devices with different types, e.g. high definition camera, network camera, door-window sensor, smart-plug, home-hub, from the same manufacturer (D-Link), to improve IoT networks performance.
• The datasets will help researchers in optimizing and analyzing network communication used by IoT devices in an IoT network, e.g. device identification [1,2], and device log prediction [3] are some of the techniques developed for IoT security by analyzing the network communication behaviour.
• The given IoT datasets can be analyzed by researchers in the context of network bandwidth and storage capability required for home or office automation since data were recorded in a separate file every day.
• The existing datasets consist of either network packets or MAC frames, while the provided IoT datasets comprise both levels of traffic traces. As such, it will help the researchers to investigate the comprehensive behaviour of IoT devices.

## 1 Data Description

**Fig. 1:**
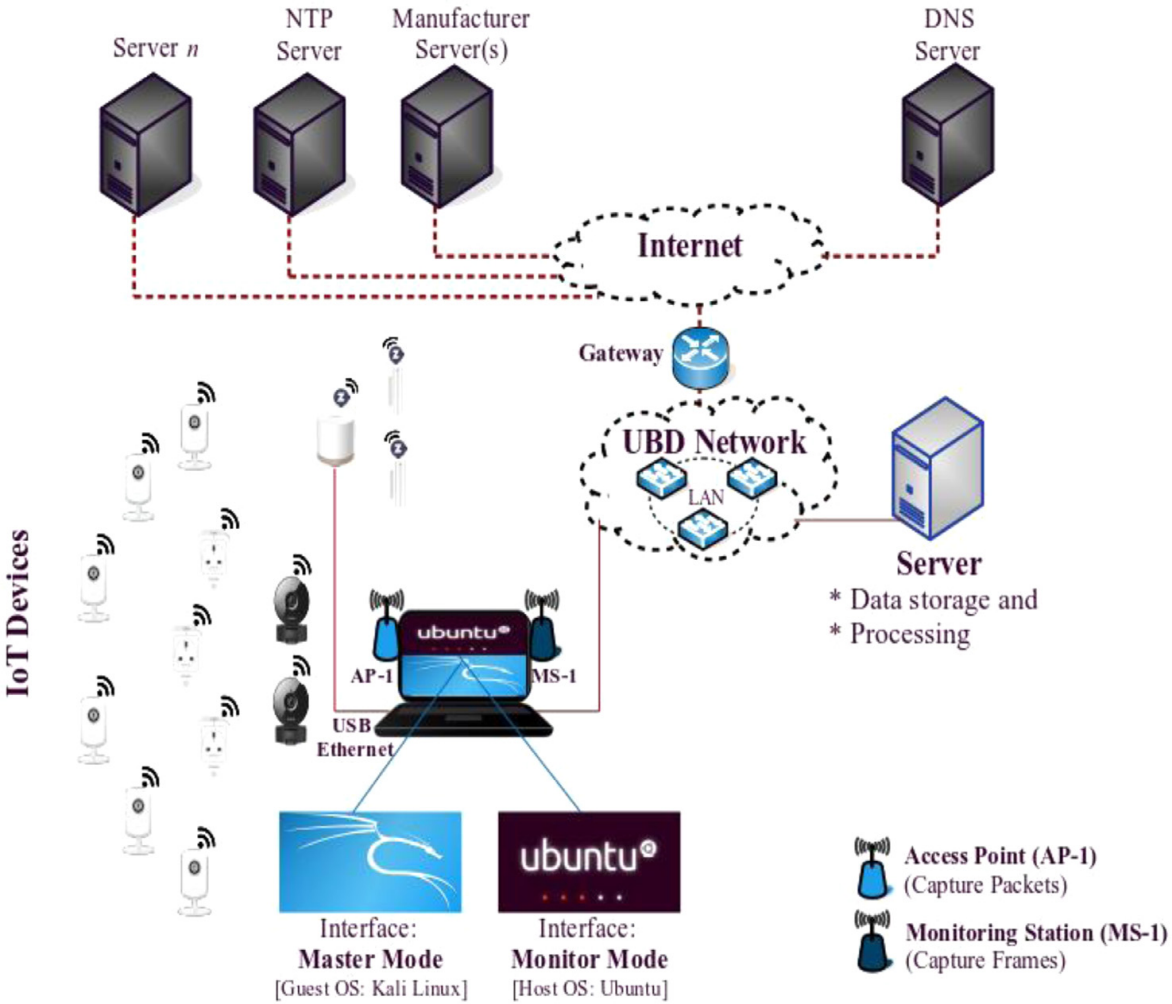
Experimental testbed design of an IoT network for network traffic traces collection.

The network traffic traces datasets consist of two levels of traffic: packet-level and frame-level, which were observed passively in the NSSP laboratory. The experimental testbed was deployed in the NSSP laboratory, located at the Faculty of Integrated Technologies, Universiti Brunei Darussalam, as depicted in Fig. 1. The datasets were collected for about five months from 9^th^ September 2020 to 10^th^ January 2021, with a similar configuration for the distinct IoT devices. Table 1 represents summary of the D-Link IoT devices datasets, and Table 2 defines typical activities of the devices, which can be found in the Mendeley Data repository (DOI: 10.17632/84cc8grtkt.1). A sample of collected network traffic traces from both datasets is presented in Table 3, along with a brief representation.

**Table 1:** Summary of the collected network traffic traces of the D-Link IoT devices.

Name	Size	No of Devices	No of Files	Instances
Network_Packets	6.0 GB	14	704	32,911,503
Network_Frames	171.4 MB	11	437	560,399

**Table 2:** Typical activities of the D-Link IoT devices.

Name	No	Activity	Power
HD WiFi Camera	02	Start-Stop video, Motion & Sound detection	Electrical
Wireless N Network Camera	06		
mydlink™ Home Smart Plug	03	Turn On & Off	
mydlink™ Home-Connected Home Hub	01	Access Point	
mydlink™ Home Door/Window Sensor	02	Sensing Door/Window Open & Close	Battery

**Table 3:** Samples of network traffic traces from the experimental testbed.

	Network Traffic Samples	
	Packet-Level	Frame-Level
File name	D-LinkDayCam1_5d-2020-12-13.pcap	D-LinkDayCam1_frame-2020-12-13.pcap
Total no of packets/frames	47,901 packets	506 frames (probe request)
Device MAC address	B0:C5:54:46:48:5D	
Inbound traffic traces	16,627 packets	–
Outbound traffic traces	31,274 packets	506 frames
Transport layer protocols	TCP – 6,539 packets, UDP – 25,023 packets	–
Other protocols	SSDP – 13,672 packets, ARP – 15,757 packets, TLSv1 – 3,235 packets, EAPOL – 463 packets	IEEE 802.11 – 506 frames
Traffic capture time	Dec 13, 2020, 00:00:03.297 AM to 23:59:55.610 PM	Dec 13, 2020, 00:11:39.510 AM to 17:46:06.587 PM
File size (bytes)	8,387,281 bytes	167,510 bytes
Traffic generation speed	≈ 34 packets (per minute)	≈ 29 frames (per hour)
Packet/Frame features	e.g. udp.srcport, udp.dstport, udp.length, udp.checksum, udp.stream, tcp.srcport, tcp.dstport, tcp.hdr_len, tcp.flags, tcp.flags.ns, tcp.flags.syn, tls.handshake.comp_method, tls.handshake.cipher_suites_length, tls.handshake.extension.len, tls.handshake.extension.type, tls.handshake, ip.hdr_len, ip.id, ip.len, ip.dsfield, ip.ttl, ip.opt.ra, ip.src, ip.opt.type.class, etc.	e.g. wlan.fc.type subtype, wlan.fc.ds, wlan.da, wlan.seq, wlan.fcs, wlan.ssid, wlan.ds.current_channel, wlan.bssid, wlan.supported_rates, wlan.extended_supported_rates, wlan.ht.capabilities.ldpccoding, wlan.ht.capabilities.width, wlan.ht.capabilities.rxstbc, wlan.tag.oui, wlan.wfa.ie.type, wla.tag.number, wps_device_name, wps.vendor_id, wps.model_number, etc.

**Fig. 2:**
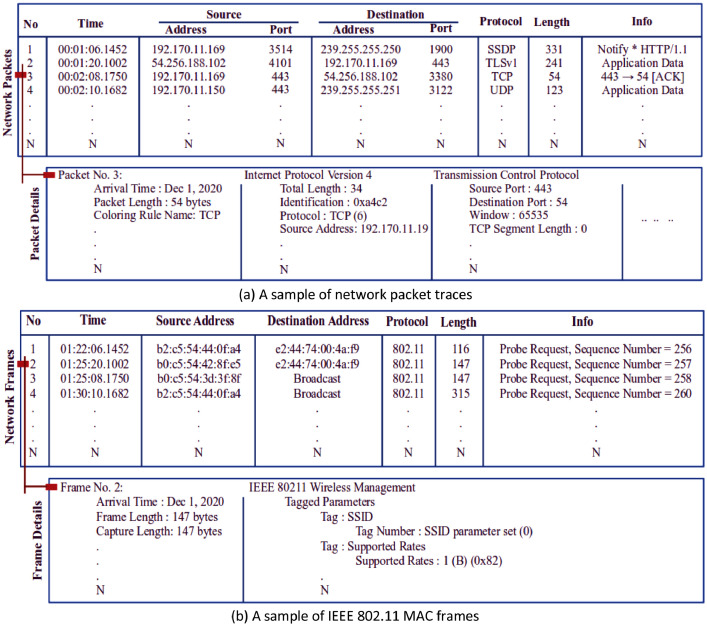
Network traffic traces samples: a) Network packets b) IEEE 802.11 MAC frames.

**Fig. 3:**
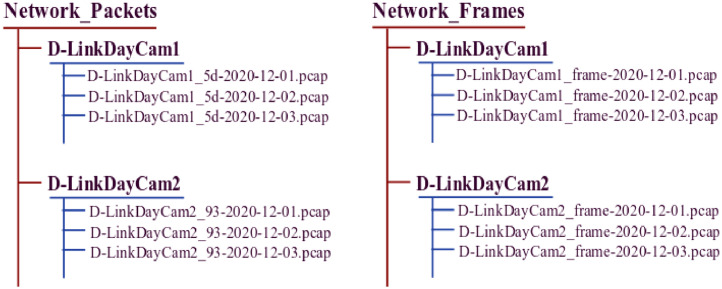
Network traffic traces files in the repository.

A network packet (consists of control information and payload) is a small amount of data which is transferred over the network during communication following a standard network protocol stack model, e.g. transmission control protocol/internet protocol (TCP/IP) model. According to the contents of the packets, different communication protocols, such as transmission control protocol (TCP), user datagram protocol (UDP), internet control message protocol (ICMP), internet protocol (IP), address resolution protocol (ARP), domain name system (DNS), hypertext transfer protocol (HTTP) etc., are used to facilitate data transmission, as depicted in Fig. 2(a). The Network_Packets dataset recorded all these protocols information by observing Ethernet and WiFi interfaces. Table 1 shows that the network packet dataset include a total of 32,911,503 packets or instances. The minimum number of instances in a file is 521, and the maximum number is 229,616. There is a total of 704 files in the repository, and all the files are arranged according to the individual device name except for D-LinkDoorSensor (two devices), as shown in Fig. 3. However, D-LinkDoorSensor traffic traces were recorded (as indirect traffic) along with D-LinkHomeHub traffic traces.

In the experiment, each file was recorded in a pcap file format and the individual file was named corresponding to the device name with the last octet of the device MAC address and recorded date, e.g. D-LinkDayCam1_5d-2020-12-01.pcap. A pcap (or packet capture) file is a data file of captured network traffic traces, which contains network communication information for analysis of network characteristics. It commonly uses some applications to capture, analyze, and open pcap files, such as Wireshark, WinDump, tcpdump, etc. Subsequently, the pcapfix tool may then be utilized to repair corrupted pcap files. In a network, packet size varies depending on the protocol used for communication. Therefore, individual file size changes dynamically based on the network activities of the devices. The packet-level dataset comprise of all inbound and outbound network traffic traces, and it was captured every day in a separate file. The network frame dataset (Network_Frames) contains probe request frames of the IEEE 802.11 MAC frame, which is the subcategory of management frames. The dataset was recorded on both associated and unassociated states of devices (station or client) communication frames. Wireless connectivity enabled devices exchange probe request frames sporadically in a wireless local area network (WLAN) for network scanning, to establish a connection with a nearby access point.

A number of information elements (IEs) tags, as shown in Fig. 2(b), such as service set identifier (SSID) parameter set, supported rates, extended supported rates, high-throughput (HT) capabilities, and vendor-specific, are embedded in a probe request frame depending on the individual device configuration and capability. Table 1 shows that there is a total of 560,399 probe request frames recorded from the D-Link IoT devices, while the total size of the frame dataset is 171.4 MB. The name of each file was recorded in the data repository according to the individual device name and captured date, such as D-LinkDayCam1_frame-2020-12-01.pcap, with the files stored in a pcap file format.

## 2 Experimental Design, Materials and Methods

An IoT network was deployed in the NSSP laboratory at UBD, which consisted of 14 different IoT devices from the same manufacturer. All these devices were connected with access points to get required services from the Internet, while access points (e.g. AP-1, HomeHub) were connected to the UBD local area network and UBD network associated with the Internet over the gateway. In the laboratory, IoT devices were located distinct position within the wireless (e.g. WiFi or Z-Wave) range. The experimental design of an IoT network for the data collection process is depicted in Fig. 1.

The experimental testbed comprised of 14 D-Link IoT devices [4], to collect network traffic traces in both packet-level and frame-level. Details of the devices are listed in Table 4, whilst Table 5 presents the individual device model's features in brief. A laptop was used to setup access points with a WiFi adapter and an external Ethernet adapter connected to the system, and a local server was utilized to store all the captured network traffic traces. The access points were configured on a laptop running Kali Linux (2020.3) as a guest operating system (OS) over the VMware Workstation 15 Player (15.5.2) along with software packages hostapd and dnsmasq, and Ubuntu (18.04) as the host OS. Additionally, a WiFi adapter was connected with the system (guest OS) to use as a WiFi interface on master mode.

**Table 4:** List of D-Link IoT devices.

Device	Name	Model	MAC Address	Connectivity
D-LinkCam1_a4	HD WiFi Camera	DCS-936L	b2:c5:54:44:0f:a4	WiFi
D-LinkCam2_11			b2:c5:54:44:0f:11	
D-LinkDayCam1_5d	Wireless N Network Camera	DCS-930L	b0:c5:54:46:48:5d	WiFi/Ethernet
D-LinkDayCam2_93			b0:c5:54:3d:3e:93	
D-LinkDayCam3_8f			b0:c5:54:3d:3f:8f	
D-LinkDayCam4_a6			b0:c5:54:42:8f:a6	
D-LinkDayCam5_e5			b0:c5:54:42:8f:e5	
D-LinkDayCam6_88			b0:c5:54:42:8f:88	
D-LinkSmartPlug_55	mydlink^TM^ Home Smart Plug	DSP-W215	c4:12:f5:16:40:55	WiFi
D-LinkSmartPlug_6e			c4:12:f5:16:40:6e	
D-LinkSmartPlug_3b			c4:12:f5:16:40:3b	
D-LinkHomeHub	mydlink™ Home-Connected Home Hub	DCH-G022	a0:ab:1b:7c:8b:48	WiFi/Z-Wave/Ethernet
D-LinkDoorSensor_1	mydlink™ Home Door/ Window Sensor	DCH-Z112	–	Z-Wave
D-LinkDoorSensor_2

**Table 5:** Features of the D-Link IoT devices.

Model	Description
DCS-936L	The HD Wi-Fi Camera is a high quality wide-angle lens surveillance camera, including night vision, motion and sound detection systems. The built-in infrared (IF) light allows viewing at night time up to 5 meters using this camera. This WiFi-enabled HD camera can be configured using the mydlink™ app to control, e.g. trigger push notification or email with snapshots or video clips according to sound and motion detection mechanisms, and monitor over the network. It has a micro-SD card slot to facilitate data storing capacity on the camera.
DCS-930L	The Wireless N Network Camera is a surveillance tool for monitoring small areas, such as home, office, in daylight (or electric light) over the network. This device integrates with motion and sound detection systems to provide additional services automatically according to the user's preferences. It can be connected to the network either using WiFi or Ethernet interfaces, hence, the user's of a device can visualize camera status anytime from anywhere by using compatible hardware (e.g. smartphone, personal computer, laptop) and software (e.g. mydlink™ Lite).
DSP-W215	The mydlink™ Home Smart Plug allows a device user's to control electronic appliances (e.g. washing machine, table lamp, electric kitchen equipment) over the network to perform on/off operations. This WiFi-enabled device works with suitable hardware (e.g. smartphone) and software (e.g. mydlink™ Home app), which helps to setup auto on/off scheduling, monitor usage of energy and configure safety alerts. The integrated thermal sensor of the smart-plug device turns off overheating sockets automatically for safety.
DCH-G022	The mydlink™ Home-Connected Home Hub acts as an access point (e.g. router), which empowers connected various smart devices either using WiFi, Ethernet, or Z-Wave technologies to provide network services. By using the mydlink™ app on compatible devices, e.g. smartphone, tablet, a user can control and monitor all the connected devices over a network.
DCH-Z112	The mydlink™ Home Door/Window Sensor alerts or notifies a user's of the device over the network when a door or window is opened or closed. This device can trigger other devices activities if devices are connected in the same network according to a user's configuration utilizing the mydlink™ app.

An external Ethernet adapter was connected with the host OS to configure the Ethernet interface typically available on an AP. All the IoT devices were setup using a smartphone to connect with the access points, and tcpdump [1,5] utility was used on the system to capture network packet traces.

Henceforth, host OS was configured to use a WiFi interface on monitor mode to collect IEEE 802.11 MAC frame traces using tcpdump. All the captured network traffic traces (WiFi and Ethernet) were stored in pcap file format. *C*ron job (a daemon) utility was used to automate data collection and storage processes, with these processes starting every day at 12:00 AM local time-zone using shell scripts.

## Ethics Statement

The authors state that they have no ethical issue in this work since network trace traces were collected in a control experiment using open source software, manuals, and publicly available referenced articles in the laboratory.

## Credit Author Statement

**Rajarshi Roy Chowdhury:** Conceptualization, Methodology, Investigation, Writing - Original draft preparation; **Sandhya Aneja:** Supervision, Conceptualization, Writing - Review & Editing; **Nagender Aneja:** Conceptualization, Methodology; **Pg Emeroylariffion Abas:** Writing - Reviewing and Editing, Validation.

## Declaration of Competing Interest

The authors declare that they have no known competing financial interests or personal relationships which have influenced the work reported in this paper.
